# Coal fly ash as a resource for rare earth elements

**DOI:** 10.1007/s11356-015-4111-9

**Published:** 2015-01-24

**Authors:** Wojciech Franus, Małgorzata M. Wiatros-Motyka, Magdalena Wdowin

**Affiliations:** 1 Department of Geotechnics, Civil Engineering and Architecture Faculty, Lublin University of Technology, Nadbystrzycka 40, 20-618 Lublin, Poland; 2 Department of Chemical and Environmental Engineering, Faculty of Engineering, University of Nottingham, NG7 2RD Nottingham, UK; 3 The Mineral and Energy Economy Research Institute of the Polish Academy of Sciences, Wybickiego 7, 31-261 Kraków, Poland

**Keywords:** Rare earth elements, Coal fly ash utilisation

## Abstract

Rare earth elements (REE) have been recognised as critical raw materials, crucial for many clean technologies. As the gap between their global demand and supply increases, the search for their alternative resources becomes more and more important, especially for the countries which depend highly on their import. Coal fly ash (CFA), which when not utilised is considered waste, has been regarded as the possible source of many elements, including REE. Due to the increase in the energy demand, CFA production is expected to grow, making research into the use of this material a necessity. As Poland is the second biggest coal consumer in the European Union, the authors have studied different coal fly ashes from ten Polish power plants for their rare earth element content. All the fly ashes have a broadly similar distribution of rear earth elements, with light REE being dominant. Most of the samples have REE content relatively high and according to Seredin and Dai (Int J Coal Geol 94: 67–93, 2012) classification can be considered promising REE raw materials.

## Introduction

Despite growing importance of renewable energy sources (i.e., geothermal, wind and solar) (Bertani 2010; Lund et al. 2011; Tomaszewska and Pająk 2012; Tomaszewska and Szczepański 2014; Dinçer 2011), coal’s share of the global energy mix continues to rise and by 2017 will come close to surpassing oil as the world’s biggest energy source (IEA 2012). With the global energy demand growing, coal is seen as a reliable and secure energy source and many countries are increasing its consumption (IEA 2014). Consequently, the global coal fly ash production increases and there is a pressing need of its recycling and utilisation (Yao et al. 2015; Mattigod 2003). Although, there is a number of possible fly ash applications, on average, only 25 % is utilised, the rest is considered a pollutant and disposed of as waste (Blissett and Rowson 2012; Sommerville et al. 2013). It is thought that coal combustion represent a large-scale mobilisation of all naturally occurring elements in a magnitude that is comparable to the rates of mobilisation and transport that occur as part of natural geochemical cycling of elements (Bertine and Goldberg 1971; Hendrick 1995). Hence, the recent research onto coal fly ash (CFA) has been focused on its novel applications (Blissett et al. 2014). Although there are many publications in regard to fly ash utilisation, there are only very few looking into its potential as a resource of REE, and even fewer investigate coal-biomass fly ashes for this purpose (Heebnik 2011; Blissett et al. 2014). In addition, most of investigated fly ashes come from exploratory coal samples that have been laboratory ashed for analytical investigations, not from coal fly ash stockpiles (Blissett et al. 2014). Because the use of biomass as a co-firing fuel in power plant is increasing, there is an increase in a production of ‘new kind’ of fly ashes, which have different properties from coal fly ash and consequently often are not suitable for many common applications (e.g., in cement and concrete). Hence, it is important to investigate alternative application of both coal and coal–biomass fly ashes. This work aims to study both types of fly ash as a potential source of REE, by investigating samples from ten coal-fired power plants in Poland. Rare earth elements are a group of 17 elements (15 lanthanides plus yttrium and scandium), which are particularly used in many new technologies such as fuel cells, green energy devices, hi-capacity batteries, magnets for wind power generation, to name but a few (Chen 2011; Całus-Moszko and Bialecka 2013; Hoenderdal et al. 2013).

In natural environment, REE are a group of elements with a high potential as process indicators in the system rock or soil as well as water (Grawunder et al. 2014). They can be classified (geochemically) into the following: light earth elements (LREE — La, Ce, Pr, Nd and Sm), medium (MREE — Eu, Gd, Tb, Dy and Y) and heavy (HREE — Ho, Er, Tm, Yb and Lu) (Blissett et al. 2014). However, Seredin and Dai (2012) devised a new REE classification which takes into account current market trends and is related to the likely supply and demand forecast over next few years. This classification divides REE into three groups: critical (Nd, Eu, Tb, Dy, Y and Er), uncritical (La, Pr, Sm and Gd) and excessive (Ce, Ho, Tm, Yb and Lu). Naturally, this classification makes an evaluation of the REE distribution more relevant to the industry (Blissett et al. 2014). It needs to be noted that the commercial importance of REE is not reflected in the amount in which they are used, but in the fact that they are crucial in a wide range of applications including key technologies for the clean and sustainable energy (Alonso et al. 2012; Du and Greadel 2013; Seredin et al. 2013). REE resources are not that rare (deposits have been identified in 34 countries—according to Chen (2011)), but because their mining and production are not cheap and can be associated with significant environmental impact (radioactivity of elements associated with them—uranium and thorium as reported by BGS-NERC 2010), they have low substitution and low recycling rates, most of world’s economies/countries including the European Union (EU) depend highly on the import (Massari and Rubeti 2013). In recent years, majority of countries which produce and export REE, have reduced their export to the EU in order to protect their national downstream industries. For example China, which is world’s main producer and exporter of REE (c.a. 97–98 %) has progressively increased export taxes on them and restricted their export rates (a decrease of about 40 % between 2009 and 2010) (Alonso et al. 2012; Massari and Ruberti 2013). As there is a gap between REE’s growing demand and decreasing supply, it is extremely important to look for the alternative resources.

Kertis and Yudowich (2009) estimated that CFAs contain 445 ppm of rare earth elements (REE) on an average global basis. and as Seredin and Dai (2012) noted, ‘if the average REE content in coal ashes, based on analyses of numerous samples, is comparable to some conventional ores, the wastes of coal combustion could and should be considered as possible source of these materials.’

Among all REE, lanthanum, europium, erbium and neodymium are considered the most important for the new technologies (Całus-Moszko and Białecka 2013). For example, lanthanum, which is the second most common element among all REE and in the natural environment is present in greater amount than silver and lead, is essential component in hybrid cars, while europium has been used in ultraviolet LED devices, erbium in lasers used for medical operations, neodymium—in magnets. Additionally, REE have been used in fertilisers (Pang et al. 2002).

As most of investigations into coal fly ash were concentrating on their use in the concrete/cement industry or for the production of synthetic zeolites (Querol et al. 2002; Franus et al. 2014; Wdowin et al. 2014), the investigation into CFA as a potential source of REE is considered a new research area. However, it seems to attract more and more attention and investment. REE extraction process from fly ash is considered to be far less intensive than that of mining a raw product. In addition, CFAs contain a full range of REE, whereas most mines have only a few varieties of these elements. Due to these reasons, many companies are exploring methods to extract REE from coal fly ash and investigating whether coal fly ash processing can become an economical and environmentally friendly alternative to traditional mining (Mayfield and Lewis 2013). For example, Neumann Systems reported that their process has successfully extracted more than 60 % of the available metals (including 14 rare earth and strategic metals) from fly ash samples supplied by a Colorado Springs Utilities power plant. The company estimates that it can extract about $600 worth of rare elements from every ton of coal ash and that the waste from Martin Drake plant alone can produce c.a. $49 million worth of minerals a year. Moreover, to dissolve REE out of fly ash, the extraction process uses by-products of scrubbing emissions (sulphuric and nitric acids) which is considered an additional benefit (Currie 2012).

For the complete evaluation of the coal fly ash as a raw source of REE, a number of parameters should be taken into account, including the possibility of beneficiation and simplicity and cost of hydrometallurgical recovery and various environmental issues. However, for the initial evaluation, data on REE individual contents within the investigated coal fly ash may be adequate (Seredin and Dai 2012).

## Materials and methods

Twelve different CFAs were obtained from ten coal-fired power plants in Poland. As seen in Table 1, nine ashes were derived from the combustion of bituminous coal among which two were from a bituminous coal/biomass blend, while the rest of the ashes were derived from lignite coal combustion.

**Table 1:** Sample list

No. of sample	Combustion type
Fly ashes from bituminous coal combustion	
1	Pulverised coal combustion
2^a^	Pulverised coal combustion
3^a^	Pulverised coal combustion
4	Pulverised coal combustion
5	Pulverised coal combustion
6	Pulverised coal combustion
7	Pulverised coal combustion
8	Pulverised coal combustion
9	Fluidised bed combustion
Fly ashes from lignite coal combustion	
10	Fluidised bed combustion
11	Pulverised coal combustion
12	Pulverised coal combustion

^a^Biomass co-firing

Morphological and mineralogical analyses were performed by the means of SEM-EDS and XRD, respectively. Chemical analyses were performed by the means of ICP-MS and ICP-EA and LOI. Although the study of rear earth elements is the primary objective of this work, trace and other elements (including the hazardous elements) of fly ashes have been investigated to see if there is a possible correlation between them and REE.

## Results and discussion

### Mineralogical and morphological characteristics

**Fig. 1:**
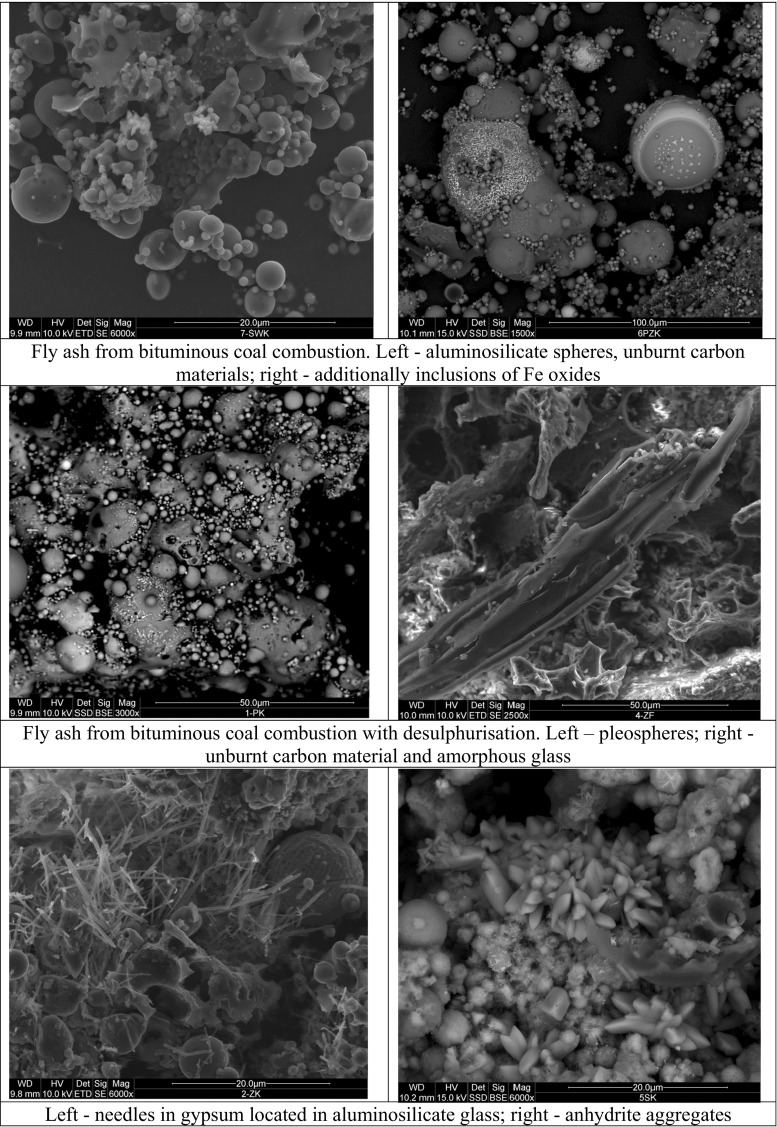
SEM microphotographs of fly ashes from bituminous coal combustion

**Fig. 2:**
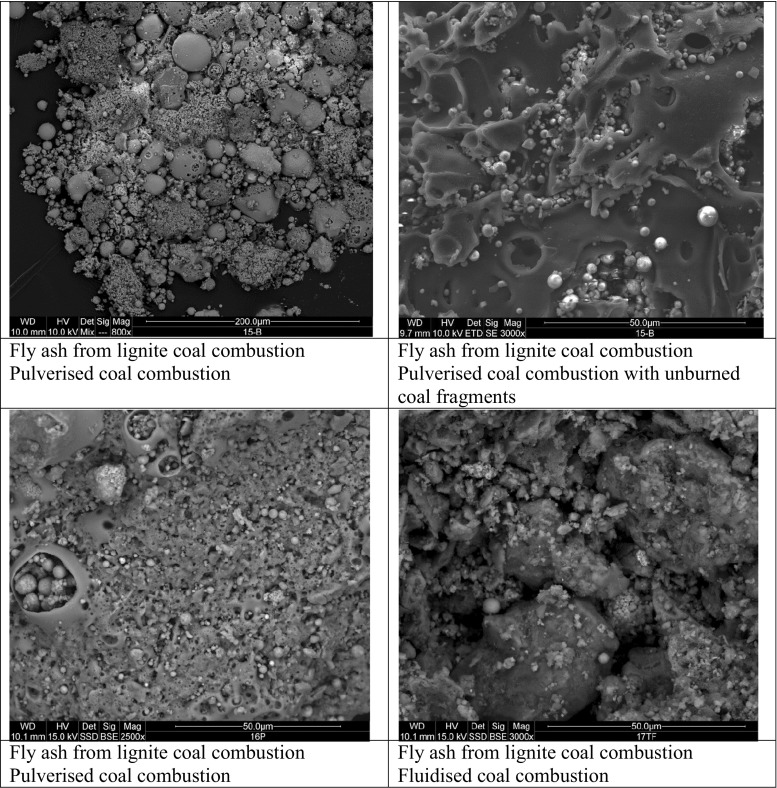
SEM microphotographs of fly ashes from lignite coal combustion

It is well established that the morphology of a fly ash particle is controlled by combustion temperature in the boiler and cooling rate in the post combustion zone (Kutchko and Kim 2006). As expected, despite the variety of fly ash samples, the mineralogical and morphological studies (SEM-EDS and XRD) confirmed that all tested fly ashes consist mainly of amorphous and cryptocrystalline aluminosilicates (Franus et al. 2014; Wdowin et al. 2014). Although particles in irregular shapes are dominant structures, some cenospheres and fractured spheres with mineral intergrowths and agglomerations inside them are also present (Figs. 1 and 2).

**Fig. 3:**
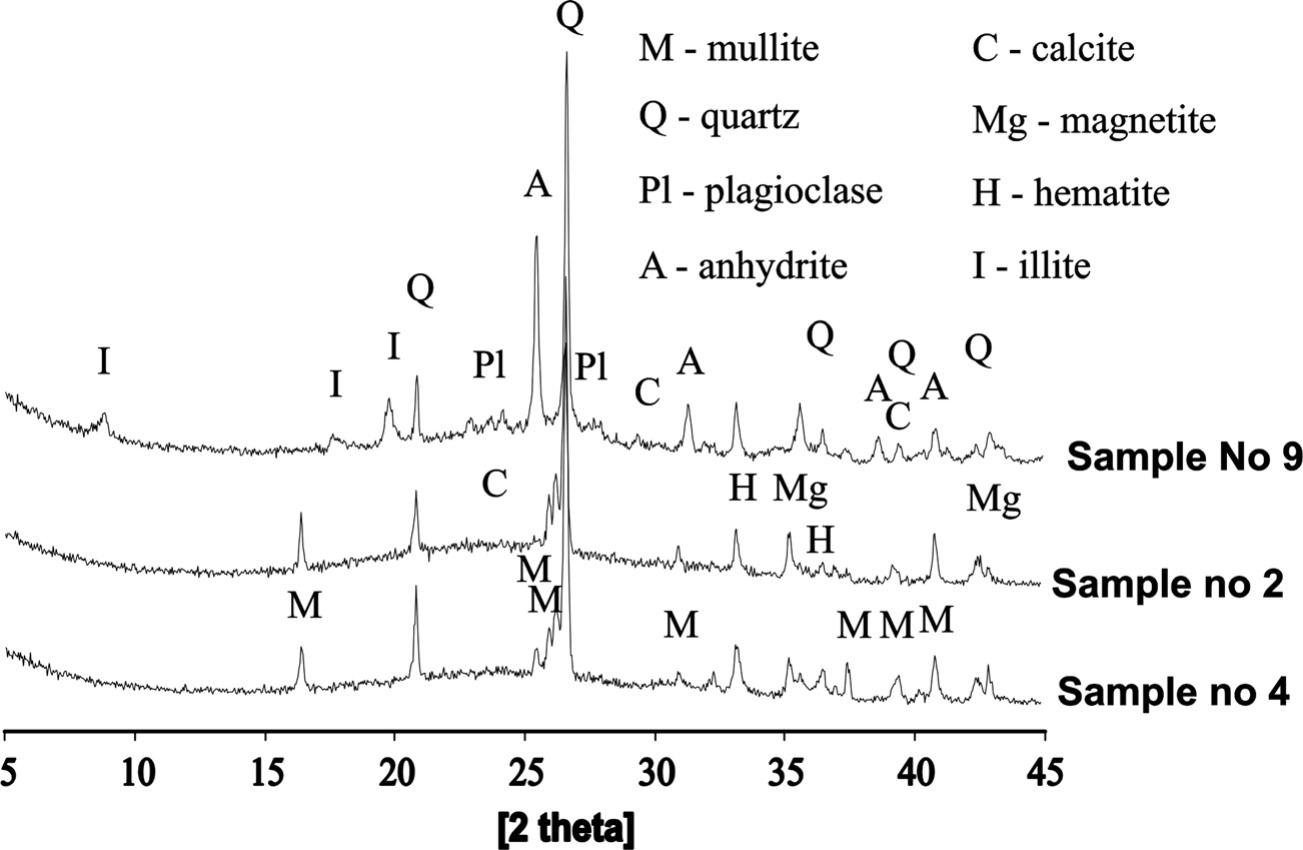
Examples of XRD diffraction patterns of fly ashes from bituminous coal combustion

In case of fly ashes from bituminous coals, XRD studies (Fig. 3) showed the presence of mullite and a lesser amount of quartz, unburned carbon, iron oxides (hematite and magnetite—frequently present as ingrowths on the glassy aluminosilicate spheres), spinel, goethite and pyrrhotite (Franus 2012). The sizes of the individual particles observed in this study ranged from less than 1 μm to even up to c.a. 500 μm.

For the samples from bituminous coal combustion, where desulphurisation process (lime added to the boiler) took place (samples 9, 10, 11 and 12), sulphur group minerals such as anhydrite (in the form of rosette aggregates) and gypsum (needle-like forms) with calcite were observed (Fig. 1). The aggregates of small amount of illite group minerals were noted in the samples obtained from fluidise combustion processes.

**Fig. 4:**
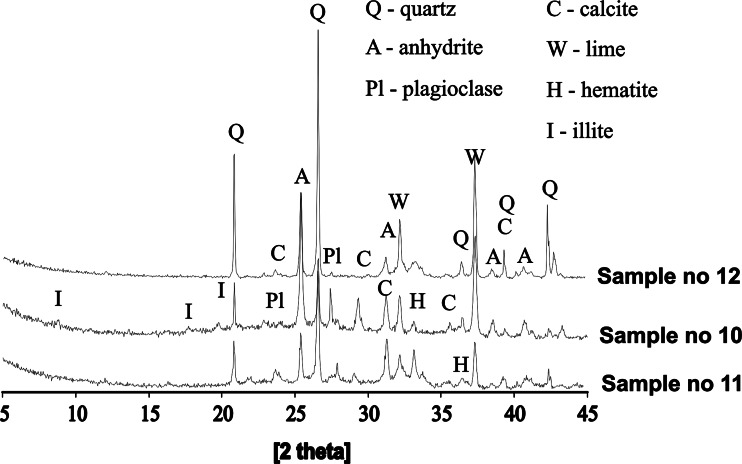
XRD diffraction patterns of fly ashes from lignite coal combustion

Fly ashes from lignite coal combustion had the same mineralogical content as fly ashes from the bituminous coals combustion (except for mullite (Fig. 4)), but differed in morphology—they appeared to be more amorphous. Similarly to the fly ashes from bituminous coal, some spherical forms with agglomerations of small particle inside them were also noted. Majority of those aggregates had a diameter of below 50 μm. Additionally, significant contents of unburnt carbon material (variety of irregularly shaped frequently porous particles in sizes up to c.a. 1 mm) were present.

It was also observed that the samples from coal/biomass blend (samples 2 and 3) and those from the fluidised combustion (samples 9 and 10) were more amorphous than the rest fly ash samples.

### Chemical analyses

It is well established that the chemical composition of coal fly ash depends primarily on the chemistry of the source coal and the combustion process. As ash is derived from the inorganic minerals in the coal such as quartz, feldspars, clays and metal oxides, the major elemental composition of coal ash is similar to the composition of a wide variety of rocks in the Earth's upper crust (EPRI 2009). Therefore, oxides of silicon, aluminium, iron and calcium contain more than 90 % of the mineral component of typical coal fly ash. Minor elements (including magnesium, potassium, sodium, titanium and sulphur) consist of c.a. 8 % of the mineral composition. Whereas the trace elements such as arsenic, cadmium, lead, mercury and selenium, together make up less than 1 % of the entire composition. All of these is consistent with the results from the chemical analyses as seen in Table 2, which shows the quantitative content of main fly ash compounds (expressed as oxides) for all samples. As expected, silicon oxide (SiO_2_) is the dominant compound for the all tested fly ashes, while the aluminium oxide is the second common oxide. In the lessen amount oxides of iron, manganese and calcium are also present. As predicted, the lignite coal fly ashes (samples 10, 11 and 12) have much higher CaO contents (23–30 %) than those derived from bituminous coal (1.1–7.6 %).

**Table 2:** Quantitative content of main fly ash compounds (expressed as oxides) (in wt%)

No of sample	SiO_2_ (%)	Al_2_O_3_ (%)	Fe_2_O_3_ (%)	MgO (%)	CaO (%)	Na_2_O (%)	K_2_O (%)	TiO_2_ (%)	P_2_O_5_ (%)	MnO (%)	Cr_2_O_3_ (%)	LOI (%)	TOT/C (%)	TOT/S (%)	SUM (%)
1	53.25	26.67	5.98	2.29	2.88	0.74	2.82	1.15	0.47	0.07	0.03	3.40	2.55	0.26	99.76
2	53.24	26.14	6.08	2.35	3.05	0.79	2.87	1.05	0.54	0.06	0.03	3.60	3.36	0.18	99.82
3	50.84	25.65	6.07	2.49	4.03	0.83	2.58	1.12	0.45	0.08	0.03	5.60	3.67	0.28	99.79
4	52.18	23.02	8.88	3.66	5.10	0.82	2.73	1.02	0.31	0.14	0.02	1.90	1.82	0.49	99.80
5	52.32	26.26	6.19	2.42	2.88	1.05	3.00	1.06	0.45	0.09	0.02	3.90	3.89	0.24	99.66
6	52.12	32.19	5.17	1.29	1.16	0.49	2.87	1.38	0.43	0.02	0.04	2.60	1.42	0.19	99.78
7	49.74	27.62	5.43	2.85	3.80	1.29	3.34	1.13	0.91	0.06	0.03	3.50	3.06	0.17	99.72
8	46.51	20.86	7.62	3.29	4.56	0.99	2.28	0.88	0.37	0.11	0.02	12.30	8.58	0.36	99.81
9	32.21	16.88	7.53	3.29	7.58	1.13	1.82	0.76	0.42	0.09	0.02	28.00	25.54	2.57	99.74
10	35.70	26.57	4.52	1.72	23.44	1.46	1.10	2.22	0.18	0.03	0.03	2.90	0.69	2.99	99.88
11	42.63	17.74	4.61	1.17	29.45	0.16	0.19	1.20	0.10	0.04	0.02	2.40	1.30	1.63	99.72
12	51.35	3.97	6.30	5.94	29.90	0.15	0.22	0.61	0.18	0.34	0.01	0.50	0.14	2.83	99.47

As far as the class of ashes is concerned, materials from bituminous coal combustion (except samples 8 and 9) can be classified as Class F (SiO_2_ + Al_2_O_3_ + Fe_2_O_2_ > 70 %) according to The American Society for Testing and Materials (ASTM) (Blissett and Rowson 2012). Sample 9 has a very high content of unburned coal (>25 %) and LOI (28 %), while sample 8 slightly too high LOI (>12 %), which exclude these materials from this category. Very high unburned carbon in sample 9 is most likely due to the coal burning conditions, which evaluation is beyond scope of this paper. Based on the same classification (by ASTM), ashes from lignite coals (samples 10–12) are Class C (SiO_2_ + Al_2_O_3_ + Fe_2_O_2_ in the range of 50–70 %).

According to a new classification proposed by Vassilev and Vassileva (2007), which groups the main bulk oxides together into four tier classification system, samples 1–8 can be classified as Sialic (SiO_2_ + Al_2_O_3_ + K_2_O + TiO_2_ + P_2_O_5_ > 77 %; CaO + MgO + SO_3_ + Na_2_O + MnO < 11.5 %; Fe_2_O_3_ < 11.5 %), whereas samples 9–12 are classified as Calsialic (SiO_2_ + Al_2_O_3_ + K_2_O + TiO_2_ + P_2_O_5_ < 89 %; CaO + MgO + SO_3_ + Na_2_O + MnO > 11.5 %; Fe_2_O_3_ < 11.5 %).

**Fig. 5:**
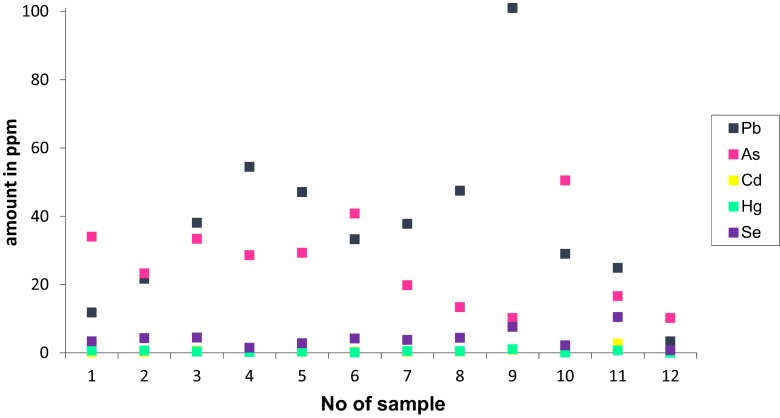
The most hazardous trace elements in tested fly ash

The detailed trace element contents for all samples are presented in Table 3 and in Fig. 5. Among the most hazardous trace elements (Pb, As, Cd, Hg, Se) (Kashiwakura et al. 2013), lead and arsenic are present in the greatest amounts. All the toxic elements are in broad agreement with toxic element contents with 23 European CFAs described by Moreno et al. (2005). There is a clear correlation between the amount of copper and lead.

**Table 3:** Trace elements (ppm)

No of sample	Element												
	Mo (ppm)	Cu (ppm)	Pb (ppm)	Zn (ppm)	Ni (ppm)	As (ppm)	Cd (ppm)	Sb (ppm)	Bi (ppm)	Au (ppm)	Hg (ppm)	Tl (ppm)	Se (ppm)
1	7.6	30.8	11.8	39.0	29.1	34.0	0.1	5.4	0.5	1.1	0.66	0.5	3.4
2	7.4	30.0	21.7	51.0	33.0	23.3	0.2	3.5	0.7	1.1	0.65	0.5	4.3
3	10.1	50.9	38.1	101.0	50.1	33.4	0.6	4.7	0.8	0.9	0.38	0.8	4.5
4	4.4	53.1	54.5	210.0	35.2	28.6	1.4	2.9	0.5	1.6	0.23	0.9	1.5
5	8.8	48.6	47.1	81.0	35.1	29.3	0.3	4.8	1.4	1.0	0.36	1.2	2.8
6	18.4	52.5	33.3	48.0	47.4	40.8	0.3	4.7	1.3	1.9	0.1	0.9	4.2
7	9.0	55.4	37.8	63.0	35.4	19.8	0.3	5.8	1.5	1.1	0.53	1	3.8
8	4.7	53.9	47.5	92.0	37.8	13.4	0.4	2.8	0.7	0.5	0.49	0.4	4.4
9	4.1	73.2	101.0	162.0	72.1	10.2	0.9	2.1	0.8	1.6	1.05	0.7	7.6
10	5.1	46.7	29.0	52.0	62.6	50.5	0.6	0.9	0.7	1.8	0.10	1.0	2.2
11	7.1	44.2	24.9	85.0	45.7	16.6	2.7	0.6	1.1	3.0	0.71	0.2	10.5
12	1.9	12.7	3.4	11.0	19.9	10.2	0.2	0.2	0.1	1.1	0.02	0.1	0.8

Although other authors (Vamvuka and Kakaras 2011) observed that biomass fly ashes are enriched in micro-nutrients, such as Zn, Cu and Mn, no significant difference between composition of fly ash from coal and biomass co-firing (samples 2 and 3) and coal firing was detected.

### REE contents and discussion

**Fig. 6:**
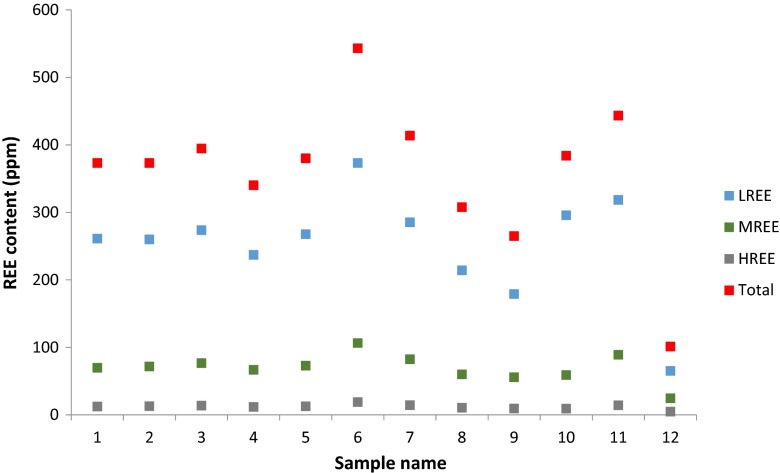
REE content in tested fly ashes

Table 4 shows individual, total and classified (as light, medium and heavy, critical uncritical and excessive groups) REE content. All samples have a broadly similar distribution of light (LREE), medium (MREE) and heavy (HREE) REE, with light REE being clearly dominant (Fig. 6). The total REE content of all the materials except samples 9 and 12 was roughly similar to data presented on fly ashes from polish coal by Blissett et al. (2014) and similarly favourable distributions in terms of their critical element content were observed. Among the critical elements neodymium (Nd) and yttrium (Y) contents were the highest and a clear correlation between them was observed. Broadly similar ranges of Nd, Ce and Sm but much lower Y, La and Gd rates (except for the material no 12) from polish coal fly ashes were reported by Smolka-Danielewska (2010). Other critical elements although in lower concentration also seem to follow the same pattern. As chemical analyses of the burnt coals were not performed/supplied by the power plants, it is impossible to draw certain correlations between coal composition and REE; it can only be assumed that their distribution in fly ashes follow the one in coal as REE are not volatile elements (Seredin and Dai 2012). Having said that, it should be noted that the combustion process results in the enhancement of REE concentrations in the coal fly ash, several times the concentration found in raw coals all over the world, including those from Poland (Całus-Moszko and Białecka 2013).

**Table 4:** Total and classified (critical, uncritical, excessive) REE contents

	Sample no.											
Element	1	2	3	4	5	6	7	8	9	10	11	12
La	59.60	59.50	62.30	54.50	62.20	81.70	64.70	48.80	41.80	74.60	76.40	15.50
Ce	123.20	121.40	127.6	110.30	125.70	172.50	133.3	100.00	83.70	137.30	142.20	30.70
Pr	13.74	13.88	14.65	12.49	14.12	20.51	15.34	11.17	9.50	15.83	17.67	3.30
Nd	53.50	54.50	57.40	49.30	54.10	81.30	59.40	44.6	35.90	57.00	69.20	12.7
Sm	11.10	10.50	11.50	10.30	11.50	17.00	12.60	9.60	8.00	11.00	12.90	2.80
Eu	2.40	2.46	2.69	2.02	2.62	3.81	2.85	1.98	1.81	2.46	2.69	0.56
Gd	8.93	9.06	9.68	8.21	9.58	14.65	10.75	7.58	6.57	8.58	11.36	2.85
Tb	1.50	1.61	1.78	1.39	1.58	2.40	1.76	1.29	1.22	1.59	1.86	0.45
Dy	8.34	8.48	8.82	8.16	9.17	12.18	9.66	6.98	6.46	7.16	9.77	2.61
Ho	1.77	1.64	1.77	1.59	1.68	2.58	1.93	1.47	1.23	1.33	1.90	0.59
Er	4.53	4.80	5.28	4.50	4.98	7.41	5.47	4.02	3.60	3.50	5.36	1.79
Tm	0.75	0.77	0.78	0.62	0.79	1.07	0.86	0.62	0.53	0.55	0.86	0.27
Yb	4.55	4.81	5.02	4.19	4.47	6.74	5.09	3.82	3.35	3.28	5.09	1.80
Lu	0.68	0.74	0.72	0.68	0.71	1.03	0.80	0.62	0.53	0.48	0.76	0.30
Y	48.50	49.0	53.60	46.90	49.80	73.20	57.20	42.10	39.60	39.10	63.30	17.9
Sc	30.00	2.00	31.00	25.00	27.00	45.00	32.00	23.00	21.00	20.00	22.00	7.00
Critical	118.77	121.75	129.57	112.27	122.25	180.30	136.34	100.97	88.59	110.81	152.18	36.01
Uncritical	93.37	92.94	98.13	85.50	97.40	133.86	103.39	77.15	65.87	110.01	118.33	24.45
Excessive	130.95	129.36	135.89	117.38	133.35	183.92	141.98	106.53	89.34	142.94	150.81	33.66
Outlook	0.91	0.94	0.95	0.96	0.92	0.98	0.96	0.95	0.99	0.78	1.01	1.07
Critical (%)	34.62	35.39	35.64	35.62	34.63	36.20	35.72	35.47	36.34	30.46	36.12	38.26
Sum	373.09	373.05	394.59	340.15	380.00	543.08	413.71	307.65	264.8	383.76	443.32	101.12

**Fig. 7:**
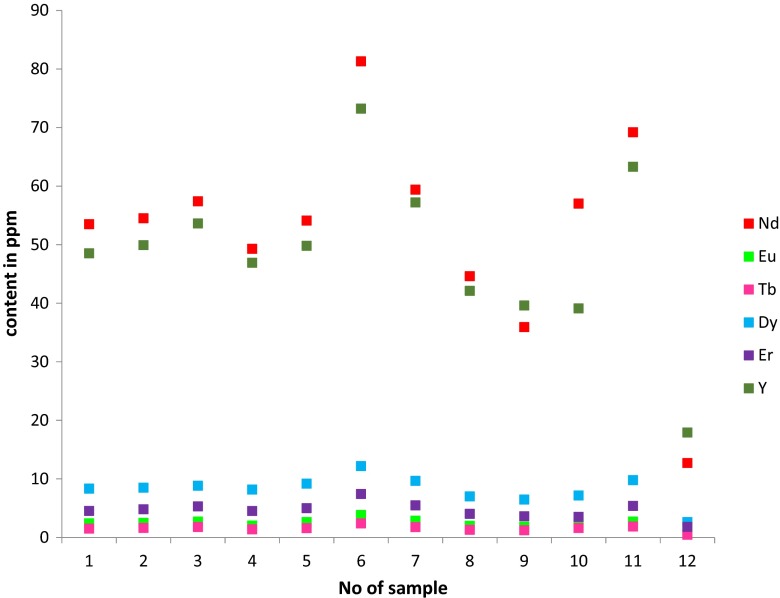
Critical REE in tested fly ashes

Following the evaluation of Seredin and Dai (2012) of REE-rich coal fly ashes based on individual REE composition, not only cumulative REE content within the fly ash is important but the proportion of their critical elements within that content and the proportion of the critical to the excessive (critical/excessive) elements expressed as an outlook coefficient are crucial. As presented in Table 4 and Fig. 7, all investigated fly ashes had critical elements contents between 30.46 and 38.26 % and outlook coefficients ranging from 0.78 to 1.07. Therefore, according to the above classification, they can be regarded as ‘promising REE raw materials for economic development’ (Seredin and Dai 2012). However, it is important to note that this statement is based on the assumption that the REE content in the whole CFA stockpile is similar to the one in the analysed sample. Having said that, it should also be noted that only three tested fly ashes (samples 6, 7 and 11) have the total REE content similar or above the world’s average (Kertis and Yudowich 2009).

Many investigations into different coals as potential source of REE focused only on the presence of rare elements in coal itself, but not in its fly ash. In general, it is believed that these elements are associated with clay-like minerals and the presence of small grains of phosphorous, sulphur and carbonate minerals (Całus-Moszko and Białecka 2013). In addition, correlations between REE presence and coal organic matter have been reported (Dai et al. 2008, 2011, 2012; Eskenazy 1987; Finkelman 1994; Seredin 1996). Moorea and Esmaeilia (2012) showed an association between occurrence of REE in coal and the presence of following chemical elements: Si, Al, Na, P, Mn, Cu, Co and Zn, while no relation with Ca and Mg was observed. Therefore, presence of REE can be associated with kaolinite, hornblende, biotite and muscovite. This was also confirmed by the work of Querol et al. (1994). The same relationships were investigated in the presented work between REE and fly ash from bituminous and lignite coal combustion. The data suggests a correlation between REE and presence of aluminium and silicon oxides (Tables 2 and 4). The similar finding was reported by Querol et al. (1995) for the coal and its combustion products, i.e., fly ashes and slags in which REE were present only in glassy aluminosilicate areas of the samples.

Obtained results were compared to findings from investigations of bituminous, bituminous-biomass, anthracite fly ash as well as soil and chondrite samples, reported by other authors (Blissett et al. 2014; Querol et al. 1995; Kabata-Pendias and Pendias 2001; Anders and Grevesse 1989) and summarised in Table 5. In comparison to other works, the analysed samples have a similar amount of REE, especially LREE, while considerably higher overall contents than soils and chondrites. Having said that, it needs to be noted that HREE contents for all compared samples (except for sample 12) are relatively similar. Hence, coal fly ashes can be considered a potential source of rare earth elements. As the initial investigations suggest that at least the three of studied samples (6, 7 and 11) have a total REE content close to the average global basis (445 ppm) and relatively low toxic elements concentrations, they should be considered for further examination.

**Table 5:** Comparison of the main results in tested fly ashes, soils and chondrites analysed by other authors

	LREE [ppm]	MREE [ppm]	HREE [ppm]
Sample 1	261.10	69.67	12.28
Sample 2	259.80	71.51	12.76
Sample 3	273.50	76.57	13.57
Sample 4	236.90	66.68	11.58
Sample 5	267.60	72.75	12.63
Sample 6	373.00	106.2	18.83
Sample 7	285.30	82.22	14.15
Sample 8	214.20	59.93	10.55
Sample 9	178.90	55.66	9.24
Sample 10	295.70	58.89	9.14
Sample 11	318.40	88.98	13.97
Sample 12	65.00	24.37	4.75
Bituminous coal fly ash^a^	240.10	0.00	68.31
Bituminous coal fly ash^b^	256.40	76.90	24.90
Anthracite fly ash^b^	359.10	92.70	28.8
Bitominous-biomass fly ash^b^	212.40	61.60	19.70
Soil (minimum REE content )^c^	6.00	1.75	8.82
Soil (maximum REE content)^c^	154.00	13.50	44.45
Chondrites^d^	1.37	1.96	0.70

^a^Querol et al. (1995)

^b^Blissett et al. (2014)

^c^Kabata-Pendias and Pendias (2001)

^d^Anders and Grevesse (1989)

As the mineral fraction in coal is subjected to many transformations during and after combustion (e.g., decomposition, volatilisation, fusion, agglomeration or condensation), there may be a significant variation of element concentration within fly ash particles (Izquierdo and Querol 2012; Jones 1995; Kukier et al. 2003). Hence, the future work should include an extensive investigation of the CFA stockpiles homogeneity (in terms of REE and other elements contents). Choosing a suitable REE recovery method will have to be tailored to the specific characteristic of each coal fly ash. As there is very limited information available regarding REE, other strategic as well as potentially hazardous element contents in the existing fly ash stock piles in Poland (and in the world), it is impossible to predict accurately which recovery process will be the most effective and what future waste streams will be generated. Those fly ash deposits which will be identified as being potentially economically viable will undergo a full chemical characterisation in order to establish which contaminants may require specialised waste handling measures. Although REE extraction and separation methods are still under development, there is already a number of techniques available (Mayfield and Lewis 2013; Meawad et al. 2010). Their efficiencies vary considerably (50–90 %) and depend on many factors including concentration of different elements in the fly ash (Arroyo et al. 2009). Hence, the full evaluation of fly ash properties and their chemical characterisation are needed in order to choose the most suitable process for each individual fly ash deposit. Furthermore, full assessment of the public health and environmental risks of pollutants generated during the process must be undertaken.

## Conclusions

With the growing energy demand and consequent increase in the use of coal (IEA 2014), the problem of coal fly ash disposal is expected to worsen (Izquierdo and Querol 2012). On the other hand, prices of strategic minerals and rare earth elements, which are essential to many advanced technologies (e.g., wind turbines, electric vehicles, permanent magnets and mobile phones), have been rising significantly due to the increasing global demands and supply shortages (Mayfield and Lewis 2013). Therefore, in addition to new mining ventures and electronic recycling schemes (Binnemans et al. 2013), many companies search for new mineral sources, among which CFA is considered a promising one (Mayfield and Lewis 2013). As extraction of REE from coal fly ash is still developing and have yet to be commercialised, there is a limited knowledge of their contents in the existing coal fly ash deposits. Hence, identification and full characterisation of current coal fly ash deposits, which are economically viable for REE (and other strategic metals) recovery is necessary.

While this initial investigation onto coal and coal-biomass fly ashes showed the variation in the total REE content between different samples, all tested materials had over 30 % of the critical REE content and an outlook coefficient >0.7, which suggests that at least the three samples (6, 7 and 11) with a total REE content close to the average global basis (445 ppm) should be considered for the further examination. Future work will include investigation of the CFA stockpiles homogeneity in terms of REE contents and an extensive chemical characterisation to determine the resources with minimal concentration of hazardous substances that require treatment and disposal. Additionally, possible extraction processes and their economic feasibility will be studied.
